# Exploring Willingness: What Drives Dialysis Withdrawal Decisions in Patients With End-stage Renal Disease? A Cross-sectional Study

**DOI:** 10.1097/jnr.0000000000000684

**Published:** 2025-06-24

**Authors:** Cheng-Pei LIN, Jung-Chi LEE, Chi-Feng PAN, Yu-Chi CHEN

**Affiliations:** 1 Institute of Community Health Care, College of Nursing, National Yang Ming Chiao Tung University, Taipei, Taiwan; 2 Cicely Saunders Institute, Florence Nightingale Faculty of Nursing, Midwifery and Palliative Care, King’s College London, London, UK; 3 Department of Nursing, Hsinchu MacKay Memorial Hospital, Hsinchu City, Taiwan; 4 Division of Nephrology, MacKay Memorial Hospital, Taipei, Taiwan; 5 Institute of Clinical Nursing, College of Nursing, National Yang Ming Chiao Tung University, Taipei, Taiwan

**Keywords:** dialysis withdrawal, disease-related symptom, palliative care, end-stage renal disease, advance care planning

## Abstract

**Background:**

Withdrawal from dialysis in patients with end-stage renal disease (ESRD) can mitigate futile treatments and facilitate early end-of-life care preparation. However, the reasons patients willingly withdraw from elective dialysis under varying disease prognoses, and the factors that influence these decisions, remain unclear.

**Purpose:**

To explore the factors influencing the willingness to electively withdraw from dialysis in patients with ESRD at different disease prognoses.

**Methods:**

This cross-sectional observational study was conducted in accordance with Strengthening the Reporting of Observational Studies in Epidemiology (STROBE) guidelines. Using convenience sampling, patients aged ≥ 45 who had regularly undergone hemodialysis for more than 6 months were recruited from a medical center and a local clinic. We used structured questionnaires and chart reviews to collect data from February to April 2021. Descriptive analysis, Pearson’s correlations, and stepwise regression were employed to assess willingness to electively withdraw from dialysis.

**Results:**

The 121 participants enrolled were of an average age of 61.09 years, had undergone dialysis for 7 years, and had a median of four comorbidities. Willingness to withdraw from dialysis increased as their disease worsened. However, more than half preferred to continue dialysis, with the number of patients decreasing from 76% to 53.7% across the disease deterioration trajectory. The participants identified nephrologists as the most influential individuals in their dialysis withdrawal discussions. Factors associated with dialysis withdrawal decisions across all hypothetical prognosis scenarios (current disease conditions, irreversible complications, and estimated survival < 6 mo) included poor quality of dialysis, lower educational level (junior high school), and better knowledge of palliative care.

**Conclusions/Implications for Future Practice:**

Willingness to withdraw from dialysis is associated with dialysis quality, educational level, and palliative care knowledge under different hypothetical prognosis scenarios. Nephrologists play a pivotal role in initiating withdrawal discussions and influencing decision-making. Health care providers should consider these factors during routine renal nursing care and identify the appropriate time to initiate advanced care planning discussions. Regular monitoring of dialysis-related symptoms and quality (measured by *Kt*/*V*) and evaluating patients’ understanding of palliative care are both essential in ESRD care. As primary caregivers, nurses play a crucial role in integrating these assessments into routine care to identify patients considering dialysis withdrawal. Collaborative efforts between nurses and nephrologists are vital to initiate timely end-of-life care discussions and preparations, improve patient-centered care, and improve end-of-life outcomes in ESRD management.

## Introduction

The prevalence of chronic kidney disease (CKD) among individuals aged 64 years and older varies between 23.4% and 35.8% worldwide. CKD is a common chronic disease in the general population with an annually rising incidence (United States Renal Data System [USRDS], 2023; Zhang & Rothenbacher, 2008). CKD progresses to end-stage renal disease (ESRD), when the kidney can no longer function independently. In recent years, the number of ESRD cases treated has substantially increased in East and Southeast Asia. Taiwan has the second highest incidence of ESRD worldwide and the highest in Asia (522 per million of population [pmp]), followed by Brunei Darussalam with 507 pmp (USRDS, 2023). Patients with ESRD are recommended to receive kidney replacement therapy or transplantation to improve their survival and quality of life. However, the percentage of living kidney transplant donors is low (Patzer et al., 2016; Tsai et al., 2022), and certain critical conditions, such as donor matching in terms of age, ethnicity, blood type, heart function, and infection control, must be aligned. Therefore, maintenance dialysis remains the predominant treatment in most countries (Thurlow et al., 2021; Tsai et al., 2022).

The results of a scoping review indicate that aging, comorbidities, dialysis-related symptoms burden, and poor dialysis quality are associated with the re-admission rate and number of emergency unit stays for patients with ESRD, both of which compromise the quality of life of patients and their families (Qazi et al., 2018). Elective withdrawal from dialysis, often requested by patients near the end of life, is a common and increasingly clinically accepted practice due to treatment intensity and disease burden (Chen et al., 2018). The 2022 Clinical Practice Guidelines for the Management of Diabetes in Chronic Kidney Disease also endorses this practice to provide better person-centered care. Providing palliative care to patients withdrawing from dialysis is urgently needed to improve their quality of life through the alleviation of disease-related symptoms, amelioration of dialysis-withdrawal–related struggles and dilemmas, fostering of hope, searching for meaning in life, and facilitation of better end-of-life preparation (Imamah & Lin, 2021; Patzer et al., 2016; Tsai et al., 2022). This is particularly relevant among the growing number of patients with ESRD worldwide (USRDS, 2023) and especially in Taiwan, where more than half of patients receiving dialysis were over 65 years old (> 90% of these receive hemodialysis rather than peritoneal dialysis), have a median estimated survival of 1.6–4.6 years, and live with an average of more than three comorbidities (National Health Research Institutes & Taiwan Society of Nephrology, 2021).

Despite its benefits, the rate of access to palliative care among patients with ESRD before death is substantially lower than that among patients with cancer. In the United States, a study observing a cohort of 1226 patients with CKD from 2001 to 2013 revealed only 26% had received palliative care consultations within 6 months of death (Chen et al., 2018) while, according to the 2022 Annual Report on Kidney Disease in Taiwan, only some dialysis patients (16.7%) currently receive palliative care before death (National Health Research Institutes & Taiwan Society of Nephrology, 2021). Discussions on withdrawal from dialysis and palliative care utilization among clinicians, patients, and their families can be emotionally difficult, complex, and culturally challenging (Axelsson et al., 2020; Bhojaraja et al., 2021; Imamah & Lin, 2021; Yang et al., 2021). For example, in Asia, family-led communication is prevalent, sometimes leaving patients with no opportunity to express their care preferences (Lin et al., 2019; Menon et al., 2018). Health care professionals often feel a sense of giving up and guilt when supporting the decision of their patients to withdraw from dialysis (Axelsson et al., 2020). Therefore, discussions about withdrawal from dialysis rarely occur, leading to continued dialysis until patient death despite its increasingly poor efficacy and their deteriorating disease prognosis. The results of a retrospective chart review observational study corroborate this, revealing that 32% of the 233 decedents observed in Taiwan had continued receiving dialysis until death between 2014 and 2018 (Yang et al., 2021).

ESRD is frequently caused by other chronic diseases during middle age, with patients often experiencing a range of uncomfortable symptoms associated with various comorbidities (Da Silva-Gane et al., 2012; Fletcher et al., 2022). Their condition can also deteriorate rapidly due to the sudden onset of infections or the impact of other treatments, leading to physical discomfort that adversely affects dialysis efficacy. Therefore, monitoring the symptoms and quality of dialysis under nursing care is important (Fletcher et al., 2022). As there is often insufficient time for meaningful end-of-life care discussions and decision-making between patients and their families after dialysis treatments have been discontinued, initiating end-of-life care planning and palliative care interventions at the point when patients indicate a desire to withdraw from dialysis should be prioritized (Axelsson et al., 2020; Bristol et al., 2021; Chen et al., 2018). Regular monitoring of the symptoms and quality of dialysis in patients with ESRD and exploring their understanding of palliative care should be integrated into routine renal nursing care (Fletcher et al., 2022). This approach ensures timely discussions about future treatment plans. However, exploring patient perspectives and understanding the timing of initiating conversations about dialysis withdrawal are challenging (Axelsson et al., 2019; Killackey et al., 2020). To provide patient-centered care in the treatment of ESRD, health care professionals must explore patient perspectives and understand the appropriate time to initiate dialysis withdrawal conversations with the whole family.

Initiating end-of-life conversations is crucial, particularly in addressing patients’ decision-making processes and the challenges faced by their families in navigating various professional responsibilities (Axelsson et al., 2020). Previous evidence has focused primarily on experiences in Western contexts, with limited representation from other cultures (Hussain et al., 2015; Martina, Geerse, et al., 2021; O’Halloran et al., 2018). End-of-life care preparations and palliative care interventions should begin early among patients, families, and health care providers, as patient diseases may deteriorate rapidly and they may continue to experience symptoms after stopping dialysis treatments. Chen et al. (2018) highlighted the underutilization of palliative care services, noting that only a third of those withdrawing from dialysis received this care. Addressing this issue requires continuous and open communication, including family meetings from the beginning of dialysis, to facilitate discussions about dialysis withdrawal and build trust for advance care planning (Axelsson et al., 2020; Chen et al, 2018). This approach improves the decision-making process, leading to more patient-centered care, and potentially improves quality of care and patient satisfaction. However, limited evidence is available regarding the perspectives of patients, particularly middle-aged and older adults, on elective dialysis withdrawal decision-making (Martina, Geerse, et al., 2021; Qazi et al., 2018). Moreover, studies on the willingness of patients with end-stage renal disease to electively withdraw from dialysis and the factors that influence this decision are scarce. Frontline dialysis nurses must assess the willingness of their patients to continue dialysis to ensure adequate and quality care.

Considering different cultural perspectives, obtaining a more profound understanding of patient care needs and decision-making processes in managing ESRD will be crucial to providing timely interventions and appropriate palliative care referrals. Therefore, this study was designed to explore the willingness of patients with ESRD to electively withdraw from dialysis and the significant factors of influence. This exploration raised current and hypothetical health condition scenarios from a non-Western cultural perspective.

## Methods

### Design

**Figure 1:**
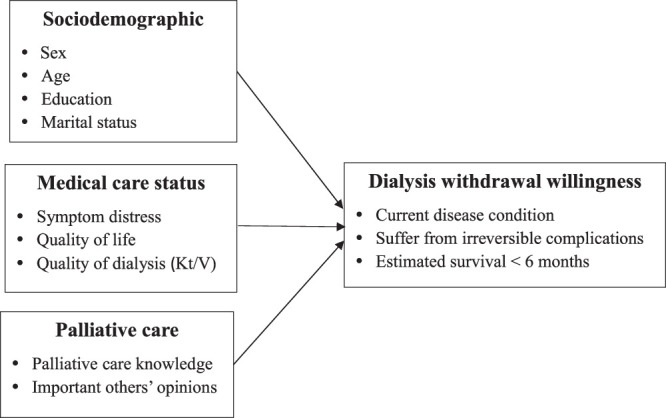
Research Framework of Influencing Factors on Willingness to Withdraw From Dialysis

Patient data were collected for this cross-sectional observational study using self-report questionnaires between February and April 2021. The associations between sociodemographic factors (sex, age, education, and marital status), clinical status (symptom distress, quality of life, and quality of dialysis), and palliative care–related factors (knowledge of palliative care and opinions of significant others) and willingness to electively end dialysis at different disease prognoses (current disease condition, suffering from irreversible complications, and estimated survival < 6 mo; Figure 1) were explored. Strengthening the Reporting of Observational Studies in Epidemiology (STROBE) guidance was adopted in reporting.

### Study Setting and Sampling

This study was conducted at the dialysis outpatient unit in a medical center and a local dialysis clinic that provides dialysis treatment under the National Health Insurance system. Convenience sampling was adopted. Patient eligibility criteria included ≥40 years of age (accounting for the majority of the population with ESRD according to the 2021 Annual Report on Kidney Disease in Taiwan [National Health Research Institutes & Taiwan Society of Nephrology, 2021]), diagnosis of ESRD, receiving regular hemodialysis for >6 months, and able to communicate in Mandarin or Taiwanese. Hemodialysis was the focus of this study, as ~92% of patients with ESRD in Taiwan receive hemodialysis therapy (National Health Research Institutes & Taiwan Society of Nephrology, 2021). The exclusion criteria included otherwise qualified individuals diagnosed with mental illness (dementia or psychiatric disorder), having impaired mental capacity, affected by verbal communication difficulties, or receiving peritoneal dialysis. Minimum sample size was estimated using G*Power 3.1.9.2 (Heinrich-Heine-Universität Düsseldorf, Germany). Assuming a medium effect size of .15, α = .05, power = .80, and nine predictors, a minimum sample of 117 participants was estimated as necessary to achieve the required statistical power, considering an expected sample response rate of 85%.

### Recruitment and Data Collection

The research team collaborated with the clinical team, briefing the dialysis unit’s lead physician, head nurse, and the local clinic on the study process, including study design, patient eligibility, data collection procedures, and outcome measures, to seek their support. Clinical staff assisted in identifying potentially eligible patients and referred them to the research team for further contact. One of the authors evaluated all potential subjects for eligibility before their regular dialysis routine, enrolling eligible and voluntarily willing patients and completing data collection. The patients completed the questionnaires after providing informed consent. A voucher (equal to NTD 100/USD 3.5) was provided to compensate for their time. In addition, data on dialysis quality and disease condition were collected by reviewing each participant’s electronic medical charts.

### Measurement

The four components in the structured questionnaire used in this study are addressed:

1. Demographic and health status: Data on sex, age, educational level, marital status, religion, employment, dialysis period, comorbidities, and quality of dialysis [hemodialysis efficiency (*Kt*/*V*)] were gathered. *Kt*/*V* > 1.2 is considered an indicator representing adequate quality for hemodialysis according to the Kidney Disease Outcomes Quality Initiative Clinical Practice Guidelines (Ikizler et al., 2020).
2. M.D. Anderson Symptom Inventory-Taiwan (MDASI-T) Form for ESRD: This inventory, based on the original MDASI, was developed for and has been tested on oncology inpatients and outpatients with good reliability and validity (Ivanova et al., 2005; Okuyama et al., 2003). This Taiwanese version inventory was adjusted and informed by Wu (2011), with items exploring symptoms of “itchy” and “lower limb weaknesses” added. The MDASI-T ESRD is a 21-item, 11-point patient self-reported Likert scale (0 = no symptoms to 10 = the highest severity imaginable) with two subscales (15 symptom severity items scored from 0 to 150, and six symptom interference items scored from 0 to 60; total score of 210). The scale has good validity and reliability, with Cronbach α scores for the symptom subscale of .85 and the interference subscale of .93 (Chang, 2003). Good reliability was also shown in this study.
3. 12-Item Short-Form Health Survey for Health-Related Quality of life assessment (SF-12): This survey comprises eight aspects, including (1) physical function, (2) physical role, (3) bodily pain, (4) general health, (5) vitality, (6) social function, (7) emotional, and (8) mental health. Scores range from 0 to 100, with higher scores indicating better quality of life (Ware et al., 1996). The psychometric properties of this scale have been previously evaluated in Spain, Sweden, and the United Kingdom with good reliability (physical component Cronbach α = .89; mental component Cronbach α = .76).
4. Palliative care knowledge scale: This survey was developed based on the authors’ clinical experiences and references (Hussain et al., 2015; Menon et al., 2018), comprising 14 questions designed to evaluate patient palliative care knowledge (10 questions on the concept of palliative care, future treatment goals, and relevant regulations scored using yes or no options; higher scores represent better knowledge of palliative care), perceived influence by others (one question scored on 4-point Likert scale; higher scores represent greater influence). This scale was assessed as providing good validity (content validity index = .95) and reliability (Cronbach α = .78) in this study.
5. Survey of willingness to withdraw from dialysis: Participant willingness to withdraw from dialysis was surveyed in three different disease prognoses, including current disease condition, suffering from irreversible complications, and estimated survival < 6 months (three questions scored on a 4-point Likert scale; higher scores represent higher willingness to withdraw).

### Ethical Considerations

This study was approved by the study hospital’s institutional review board (reference no. 20MMHIS374e). All methods were carried out in accordance with the Declaration of Helsinki. Patients completed the questionnaires after signing informed written consent, understood they could withdraw from the study at any time, and were assured their responses would be kept confidential.

### Data Management and Analysis

IBM SPSS Statistics 24.0 (IBM Corp., Armonk, NY, USA) was used for data management and analysis. Descriptive analyses of the variables were performed using percentages, means, and *SD*. Independent *t* test, one-way analysis of variance, and post hoc testing were used to examine the relationships between sociodemographic factors and willingness to withdraw from dialysis at different disease prognoses. The Pearson correlations were calculated to assess the association between dialysis quality, symptom severity, symptom interference, quality of life, and knowledge of palliative care, respectively, and willingness to withdraw from dialysis at different disease prognoses. Collinearity statistics, including variables with statistical significance informed by correlation assessment, were assessed before the stepwise regression analysis was conducted. Subsequently, assumptions of normality and independence were examined using standardized residual plots. Neither assumption was violated, and the collinearity statistics did not indicate multicollinearity among independent variables, with *p* values < .05 considered significant.

## Results

### Patient Characteristics, Dialysis-related Symptoms, Quality of Life, and Palliative Care Knowledge

Of the 140 questionnaires distributed, 121 were accepted as valid and used in subsequent analysis work. Data from 19 participants were excluded due to: (1) incomplete data collected (*n* = 9), (2) withdrawal during the study period (*n* = 9), or (3) poor condition assessed by clinicians (*n* = 1). Patient characteristics data are presented in Table 1. The average age was 61.09 years (*SD* = 9.48), the average years of dialysis was 6.58 years (*SD* = 7.33), and participants had a median of four comorbidities. Eighty-two percent were unemployed, 60% were male, 72% were married, and 62% had attained a senior high school or higher level of education.

**Table 1:** Patient Characteristics (N=121)

Characteristic	*n* (%)
Sex	
Male	73 (60.33)
Female	48 (39.67)
Age (year; *M* ± *SD*)	61.09±9.48
45–60	33 (27.27)
61–70	36 (29.75)
>70	52 (42.98)
Educational level	
Lower than junior high school	24 (19.83)
Junior high school	22 (18.18)
Senior high school	46 (38.02)
College or higher	29 (23.97)
Marital status	
No spouse	34 (28.10)
Have spouse	87 (71.90)
Religion	
No	47 (38.84)
Yes	74 (61.16)
Employment	
No	99 (81.82)
Yes	22 (18.18)
Duration of dialysis (year; *M* ± *SD*)	6.58±7.33
≤2	38 (31.40)
>2–4	32 (26.45)
>4–8	19 (15.70)
>8	32 (26.45)
Comorbidities (*M* ± *SD*)	3.79±1.49
≤2	18 (14.87)
3	39 (32.23)
4	32 (26.45)
≥5	32 (26.45)

*Note.* The median age is 61 years, the median duration of dialysis is 3.42 years, and the median number of comorbidities is 4.

The clinical status of the participants, shown in Table 2, shows data on dialysis quality, symptom status, quality of life, and knowledge of palliative care. The mean *Kt*/*V* was 1.36 (*SD* = 0.30), representing an acceptable quality of dialysis (ie, *Kt*/*V* >1.2). Reported quality of life was moderate (mean=55.79/100). In terms of dialysis-related symptoms, the participants reported an average of five symptoms with mild severity (*M*±*SD* *=*31.63±27.66). Dry mouth (*M*=5.02/10), itchy skin (*M=*4.10/10), and fatigue (*M*=3.69/10) were reported as the top 3 symptom burdens. Although symptom interference was reported as mild, they influenced physical functioning (“general activity” mean*=*2.93/10 and “walking” mean*=*2.68/10) more than emotional burden (“mood” mean*=*2.18/10). Most of the participants (60%) demonstrated inadequate knowledge of palliative care (mean=4.79/10). Nephrologists were identified as wielding the most influence on patient withdrawal decisions (66.41%).

**Table 2:** Quality of Dialysis, Symptoms Status, Quality of Life, and Palliative Care Knowledge Among ESRD Patients (N=121)

Variable	*n* (%)	Mean (*SD*)
*Kt*/*V* data	77 (63.64)	1.36 (0.30)
Number of symptoms		4.53 (3.19)
Symptom severity (MDASI-T)		31.63 (27.66)
Dry mouth	72 (59.50)	5.02 (4.39)
Itching	67 (55.37)	4.10 (4.29)
Fatigue	49 (40.50)	3.69 (3.62)
Disturbed sleep	50 (41.32)	2.38 (3.32)
Lower limb muscle weakness	46 (38.12)	2.41 (3.55)
Shortness of breath	45 (37.19)	2.85 (4.03)
Numbness/tingling	39 (32.23)	1.99 (3.28)
Distressed/upset	38 (31.40)	1.88 (3.16)
Pain	33 (27.27)	1.91 (3.44)
Lack of appetite	27 (22.31)	1.31 (2.76)
Sad	24 (19.83)	1.32 (2.75)
Drowsy/sleepy	19 (15.70)	1.00 (2.55)
Remembering things	14 (11.57)	0.75 (2.23)
Vomiting	10 (8.26)	0.58 (2.04)
Nausea	9 (7.44)	0.45 (1.73)
Symptom interference level		11.47 (15.48)
General activity	51 (42.15)	2.93 (3.95)
Walking	47 (38.84)	2.68 (3.80)
Mood	42 (34.71)	2.18 (3.39)
Work (including work around the house)	25 (20.66)	1.62 (3.39)
Enjoyment of life	22 (18.18)	1.23 (2.86)
Relations with other people	11 (9.09)	0.69 (2.33)
Quality of life (SF-12)		55.79 (21.58)
Palliative care knowledge		4.79 (2.21)
Key person to trigger the dialysis withdrawal decision		
Nephrologists	87 (66.41)	
Spouse	15 (11.45)	
Children	9 (6.87)	
Other	20 (15.27)	

MDASI-T = M.D. Anderson Symptom Inventory-Taiwan; SF-12 = 12-Item Short-Form Health Survey.

### Willingness to Withdraw From Dialysis and Related Factors Under Different Disease Prognoses

Willingness to withdraw from dialysis was found to increase with disease-related deterioration. However, more than half still expressed a preference to continue dialysis despite increasingly severe deterioration, with the proportion of patients decreasing (ranging from 76% to 53.7%; table not included). The findings indicate educational level (*p*=.05–.01), dialysis quality (*p*<.05), symptom severity (*p*<.01), symptom interference (*p*<.01), and palliative care knowledge (*p*<.01) to be statistically associated with willingness to withdraw in all three disease prognoses. Post hoc analysis highlighted those participants aged >70 years with a junior high school education, with poor dialysis quality, with severe symptoms and interference, or with better understanding of palliative care were more likely to consider withdrawing from dialysis when faced with irreversible complications or an estimated survival of < 6 months (Table 3).

**Table 3:** Factors of Influence on Dialysis Withdrawal Choices at Different Disease Prognoses (N=121)

Variable		Current Disease Condition		Suffer From Irreversible Complications		Estimated Survival < 6 mo	
	*n*	*M* (*SD*)	*t/F/r*	*M* (*SD*)	*t/F/r*	*M* (*SD*)	*t/F/r*
Sex			0.81		.00		0.34
Male	73	2.07 (0.95)		2.14 (0.98)		2.48 (1.18)	
Female	48	1.92 (0.85)		2.15 (0.95)		2.60 (1.12)	
Age (y)			2.80		4.32*		5.68**
① 45–60	33	2.27 (0.98)		2.51 (1.03)		3.06 (1.14)	
② 61–70	36	2.05 (0.95)		2.14 (0.99)		2.47 (1.16)	
③ >70	52	1.81 (0.79)		1.90 (0.82)		2.23 (1.06)	
Scheffe test					③>②		③>②>①
Educational level			3.48*		4.00**		4.53**
① Less than junior high school	24	1.71 (0.62)		1.83 (0.64)		2.17 (1.05)	
② Junior high school	22	2.50 (1.10)		2.73 (1.08)		3.23 (1.02)	
③ Senior high school	46	2.02 (0.93)		2.09 (0.98)		2.30 (1.09)	
④ College and higher	29	1.86 (0.79)		2.03 (0.91)		2.66 (1.23)	
Scheffe test			②>①		②>①		②>①, ②>③
Marital status			0.03				3.81
No spouse	34	2.03 (0.87)		2.24 (0.92)		2.85 (1.18)	
Have spouse	87	2.00 (0.93)		2.10 (0.98)		2.40 (1.13)	
Employment			0.98				3.73
No	99	2.18 (0.91)		2.36 (0.95)		2.95 (1.13)	
Yes	22	1.97 (0.91)		2.09 (0.96)		2.43 (1.14)	
Dialysis period (y)			1.10				0.64
≤2	38	2.02 (0.75)		2.11 (0.80)		2.66 (1.12)	
>2–4	32	1.97 (0.93)		2.09 (0.96)		2.44 (1.11)	
>4–8	19	2.37 (0.96)		2.42 (0.96)		2.79 (1.08)	
>8	32	1.91 (1.06)		2.15 (1.17)		2.41 (1.29)	
Comorbidities			0.83				2.15
≤2	18	1.89 (0.76)		1.89 (0.76)		2.16 (1.04)	
3	39	1.97 (0.87)		2.05 (0.94)		2.46 (1.19)	
4	32	1.91 (1.00)		2.09 (1.09)		2.41 (1.21)	
≥5	32	2.22 (0.94)		2.44 (0.91)		2.94 (1.05)	
Quality of dialysis		1.36 (0.30)	−0.19*	1.36 (0.30)		1.36 (.30)	−0.21*
Symptom severity		21.42 (22.79)	0.36***	21.42 (22.79)		21.42 (22.79)	0.42**
Symptom interference		9.91 (14.19)	0.26**	9.91 (14.19)		9.91 (14.19)	0.30**
Quality of life		29.63 (3.02)	−0.12	29.63 (3.02)		29.63 (3.02)	−0.13
Palliative care knowledge		3.98 (2.48)	0.27**	3.98 (2.48)		3.98 (2.48)	0.37**

*p < .05. **p < .01.

No multicollinearity problems were observed (tolerance *>* .1; Variance inflation: 1.10–2.93) among the factors included in the next stepwise regression model (Table 4). Educational level, dialysis quality, and patient palliative care knowledge were identified as influential factors affecting patient willingness to withdraw from dialysis in three hypothetical prognoses, with 19%–34% explanatory power [current disease conditions: *F* = 4.14, adjust *R* ^2^=.19, *p*<.01; suffering from irreversible complications: *F*=6.06, adjust *R* ^2^=.28, *p*<.01; estimated survival < 6 months: (*F*=7.72, adjust *R* ^2^=.34, *p*<.01)]. Furthermore, disease severity was considered a significant factor in different prognoses only when estimated survival was < 6 months.

**Table 4:** Regression Model for Dialysis Withdrawal Willingness at Different Disease Prognoses

Independent Variable	Current Disease Condition		Suffer from Irreversible Complications		Estimated Survival < 6 mo	
	β	*t*	β	*t*	β	*t*
Age	−0.06	−0.61	−0.13	−1.45	−0.16	−1.82
Junior high school vs. less than junior high school	0.25	2.37^*^	0.27	2.71^**^	0.23	2.47^*^
Senior high school vs. less than junior high school	0.11	0.96	0.05	0.51	−0.05	−0.46
College or higher vs. less than junior high school	0.02	0.21	0.05	0.51	0.12	1.29
Quality of dialysis (*Kt*/*V*)	−0.19	−2.21^*^	−0.14	−1.69^*^	−0.22	−2.76^**^
Symptom severity	0.16	1.19	0.15	1.14	0.19	2.50^*^
Symptom interference	0.10	0.74	0.18	1.33	0.11	0.83
Quality of life	0.01	0.07	−0.03	−0.22	−0.01	−0.09
Palliative care knowledge	0.21	2.19^*^	0.22	2.41^**^	0.27	3.03^**^
*F*		4.14^**^		6.06^**^		7.72^**^
*R*		.25		.33		.39
Adjust *R* ^2^		.19		.28		.34

* *p* < .05.

** *p*<.01.

## Discussion

In this study, a majority of the participants receiving regular maintenance dialysis exhibited a range of symptoms, with dry mouth, itchy skin, and fatigue being the most severe. Despite these symptoms, patients perceived slight interference while maintaining a moderate quality of life. Willingness to withdraw from dialysis increased as their disease deteriorated. However, more than half still preferred to continue dialysis, with the proportion of patients decreasing from 76% to 53.7%. This trend is associated with poor dialysis quality, having a junior high school education, and having better patient palliative care knowledge. Notably, nephrologists emerged as the primary influencers in initiating discussions and affecting patient decisions on dialysis withdrawal.

The demographic characteristics of participants in this study align with the 2021 Annual Report on Kidney Disease in Taiwan (National Health Research Institutes & Taiwan Society of Nephrology, 2021) and a previous systematic review conducted by O’Halloran et al. (2018), indicating that the appropriate sample was recruited. In this study, patients with ESRD in Taiwan were found to be more likely to choose dialysis withdrawal at different prognoses when they were older in age, had a junior high school educational level, had better knowledge of palliative care, experienced severe symptoms, had become frail, and had <6 months of life expectancy. This finding agrees with previous works conducted in the United States by Chen et al. (2018) and in Taiwan by Lin et al. (2020), in which one-third of patients receiving regular dialysis began palliative care consultations 6 months before their death. A similar trend was also found among advanced cancer patients in Japan, as “frailty” is the crucial indicator for beginning discussions about dialysis withdrawal (Miyashita et al., 2020). Furthermore, the scoping review of Qazi et al. (2018) corroborated that being of older age with comorbidities is a significant indicator of dialysis withdrawal due to functional declines in both physical and mental health, which increase suffering (Kurella Tamura et al., 2010; Qazi et al., 2018).

The results of this study indicate that educational level is not a direct predictor of dialysis withdrawal, but is a variable predictor based on health conditions that potentially relate to socioeconomic status and access to resources. Participants with below junior high school level education showed the lowest willingness to withdraw from dialysis across various ESRD progression scenarios. Conversely, those with a college-level education or higher showed the highest willingness to withdraw, but only when facing an estimated survival of <6 months. This may also reflect generational differences in patient autonomy. Clinically, patients with higher educational levels often explore alternative therapies or seek second opinions, especially for treatable conditions, which reduce their immediate likelihood of dialysis withdrawal. This aligns with findings by Raghupathi and Raghupathi (2020), who found that higher educational levels correlate with better health awareness and access to health care resources. In contrast, patients with lower educational levels often struggle with complex medical information and rely on family or professionals for decision-making. This lack of decision-making autonomy is particularly evident in Chinese culture, especially among elderly patients with chronic kidney disease and lower educational and socioeconomic status, for whom a firm reliance on family or professionals for health care decisions is most evident (Chen et al., 2018). This cultural context explains the lower self-reported willingness to withdraw from dialysis among the less-educated participants in this study. While educational attainment may influence decision-making, observed differences among groups are likely also affected by cultural and socioeconomic factors. Therefore, it is not feasible to attribute the decision to discontinue dialysis solely to educational level.

According to previous studies, factors such as marital status and employment are not significantly associated with dialysis withdrawal (Hussain et al., 2015; Kurella Tamura et al., 2010; Qazi et al., 2018). These discrepancies between the findings of this study and previous reviews may be explained by the limited empirical research available (only nine studies published from 2004 to 2016 in the scoping review) and the heterogeneity of the definition of dialysis withdrawal (eg, change in modality, discontinuation of dialysis for any reason by clinicians, or elective dialysis withdrawal decisions).

Symptom burdens and health-related quality of life have been identified as critical indicators guiding dialysis withdrawal and palliative care plans (Fletcher et al., 2022). However, these are not well applied in the Taiwanese context, as patients with ESRD in Taiwan often receive high-quality maintenance dialysis, resulting in mild symptom burdens and a relatively good quality of life (Wetmore et al., 2018). Although an increasing trend in participants choosing dialysis withdrawal based on prognosis deterioration was found, many still preferred to continue dialysis. Patients in Taiwan tend to receive dialysis until the end of life, and conversations regarding dialysis withdrawal may not occur (Yang et al., 2021). This phenomenon reflects the late introduction of advance care planning (ACP) to the country, which has hindered the timely exploration of end-of-life care preferences among all stakeholders (ie, patients, their families, and health care teams) due to insufficient time allowed for discussion and negotiation. In this context, nephrologists play a crucial role in encouraging patients with ESRD to consider ACP, including withdrawal from dialysis. Patients trust physicians and often ask their opinions when making difficult decisions. This phenomenon was evident in previous research on terminally ill cancer and renal disease patients in Taiwan addressing physician-provided advice and support to patients regarding the signing of “Do-Not-Resuscitate” forms and the provision of palliative care (Chen et al., 2019; Lin et al., 2020; Wen et al., 2013). A survey of 522 patients in Pakistan receiving maintenance dialysis reported a similar trend, with 80% of respondents relying on their physicians (nephrologists or family physicians) for medical information and decisions (Saeed et al., 2020). Lin et al. (2020) found that around 40% of patients and caregivers would not choose to stop dialysis during the patient’s terminal illness stage because health care providers did not initiate the conversation. The main barriers for health care providers to initiate this conversation include the lack of a dialysis withdrawal protocol, no integration of palliative care into routine ESRD care, and the lack of confidence among staff to provide symptom management to patients after dialysis withdrawal.

Patients with ESRD who undergo dialysis can prolong their lives for several years. During the long-term care process, determining the appropriate time to intervene with the ACP can be emotionally and culturally challenging. Evidence has shown the importance of considering physiological data in evaluating dialysis quality. To the authors’ knowledge, this is the first study to include dialysis quality (ie, *Kt*/*V*) as a vital indicator, distinguishing it from previous research focusing on serum albumin, serum creatinine, serum potassium, hemoglobin, and urea acid (Chen et al., 2018; Da Silva-Gane et al., 2012; Qazi et al., 2018). In agreement with prior studies, poor dialysis quality was found to correlate with longer hospital stays, burdensome symptoms, and increased patient mortality and to ultimately lead to poor quality of life (Fletcher et al., 2022; Wu, 2011). Inadequate dialysis exacerbates ESRD symptoms and complications, prompting thoughts of dialysis withdrawal (Chen et al., 2018; Hussain et al., 2015; O’Halloran et al., 2018; Wetmore et al., 2018). However, dialysis withdrawal discussions often come too late, after symptoms have already emerged. Thus, we recommend health care providers consider *Kt*/*V* as a vital laboratory marker in evaluating treatment and offer opportunities for patients with ESRD to discuss the option of dialysis withdrawal once all means for improving dialysis quality have been exhausted. Furthermore, considering *Kt*/*V* allows timely detection and anticipation of poor dialysis quality, enabling the initiation of early palliative care conversations and end-of-life care preparations.

The findings demonstrate that better palliative care knowledge in patients is associated with dialysis withdrawal willingness, a finding supported by the systematic review of Morton et al. (2010) on the views of patients and caregivers regarding treatment decision-making. Although health care professionals’ beliefs and knowledge about palliative care were not examined in this study, clarifying this will be fundamental to improving this issue. A qualitative focus group study by Axelsson et al. (2019) reported that the health care provider’s knowledge regarding palliative care is critical to facilitating the dialysis withdrawal conversation with patients. Palliative care has been shown to improve the quality of life for those who withdraw from dialysis (Bagasha et al., 2020; Menon et al., 2018). Providing accurate and timely information on palliative care facilitates patient access to services for better symptom management after dialysis withdrawal and helps mitigate the feelings of guilt experienced by family caregivers and health care professionals when supporting patient decisions to withdraw (Bristol et al., 2021).

Dialysis withdrawal decisions appear to vary significantly by race and ethnicity. A scoping review by Qazi et al. (2018) reported a higher rate of dialysis withdrawal among white individuals, highlighting the importance of considering different social roles and cultural values in these discussions. In Asian culture, individuals typically do not make independent medical decisions, but rather prefer to consult with and even transfer their autonomy to significant others, including health care providers and family caregivers (Lin et al., 2020; Martina, Geerse, et al., 2021; Wen et al., 2013). Therefore, the relational approach to autonomy, which aligns with local social norms, can be practical when making decisions about palliative care in the Asian context (Martina, Lin, et al., 2021; Miyashita et al., 2020; Wen et al., 2013). Health care providers should recognize that individual treatment preferences may sometimes be influenced by their relationships and responsibilities to others (Killackey et al., 2020; Miyashita et al., 2020). Cultural challenges to initiating discussions about dialysis withdrawal in Asia include traditional norms that prevent patients from expressing their preferences, social norms related to filiality, the aversion to depleting a patient’s hope in fighting their disease, difficulties in predicting the disease trajectory, lack of end-of-life care communication skills among health care staff, and poor understanding of palliative care with limited access (Chen et al., 2018; Martina, Geerse, et al., 2021; Qazi et al., 2018). Understanding patient willingness to withdraw from dialysis and its associated factors in different disease prognoses and cultural contexts may help in the development of tailored care interventions that promote the goal-concordant management of dialysis-related symptoms and improved outcomes. Developing a care pathway supported by the multidisciplinary care team that includes symptom management and continuous training in palliative care for health care staff is warranted (Bhojaraja et al., 2021; Martina, Geerse, et al., 2021; Menon et al., 2018; O’Halloran et al., 2018).

International consensus highlights the importance of needs-based palliative care referral rather than diagnosis-based referral (Collins et al., 2023). High-quality palliative care involves recognizing each patient’s unique and diverse needs, ensuring interventions are tailored to meet individual requirements, thus advocating for a patient-centered approach to care delivery. Therefore, it is essential to apply the critical indicators confirmed in this study (ie, *Kt*/*V* data, age group, educational level, severity level, and palliative care knowledge) to identify those needing dialysis withdrawal discussions if appropriate.

### Strengths and Limitations

This study has several strengths. First, it is a pioneering study exploring the perspectives of patients with ESRD on withdrawing from dialysis in the Asian context, particularly in light of newly enacted legislation (Patient Right to Autonomy Act in Taiwan). Second, a robust methodology was adopted that employed validated questionnaires and laboratory data to support the findings. Third, the researchers achieved a high response rate (86%) through proactive patient engagement. However, some limitations advise cautious interpretation of the results. First, using convenience sampling with a limited sample size negatively influences the generalizability of the findings. Second, as patient willingness to withdraw may be influenced by current disease trajectory, the cross-sectional research design and its reliance on hypothetical prognosis scenarios may not fully capture these changes in patient preferences regarding dialysis withdrawal and laboratory data (eg, *Kt*/*V*) over time. Longitudinal follow-ups with a cohort should be pursued in the future to address this concern. Third, data on health behaviors (eg, smoking, drug, or alcohol) and the perspectives of health care providers on dialysis withdrawal, both of which have been identified as factors of influence on the decision-making process, were not collected.

### Conclusions

This study provides significant insights into the factors influencing the willingness of patients with ESRD to withdraw from dialysis, with a particular focus on dialysis-related symptoms. The key findings reveal that dialysis quality, as measured by the biomarker *Kt*/*V*, educational level, and palliative care knowledge, play pivotal roles in the patient decision-making process. These findings underscore the need for a more nuanced approach to routine renal nursing care and palliative care practices, emphasizing the timely and effective management of dialysis symptoms and preparation for end-of-life care.

Nephrologists, supported by comprehensive assessments provided by frontline nurses, were identified as playing a critical role in initiating discussions about future care options, including dialysis withdrawal. This collaborative approach is essential in understanding and respecting patient preferences, which is a cornerstone of patient-centered care in ESRD management. By elucidating these key factors, this study contributes significantly to the existing body of knowledge in nursing and patient care for ESRD, underscoring the importance of personalized care strategies that address medical needs and consider educational and psychosocial factors. Ultimately, this research paves the way for enhancing the quality of ESRD end-of-life care and aligns with the goals of patient-centered care and improving overall patient well-being.
